# Targeting AKT/mTOR and Bcl-2 for Autophagic and Apoptosis Cell Death in Lung Cancer: Novel Activity of a Polyphenol Compound

**DOI:** 10.3390/antiox10040534

**Published:** 2021-03-29

**Authors:** Sucharat Tungsukruthai, Onrapak Reamtong, Sittiruk Roytrakul, Suchada Sukrong, Chanida Vinayanwattikun, Pithi Chanvorachote

**Affiliations:** 1 Doctor of Philosophy Program in Interdisciplinary Pharmacology, Graduate School, Chulalongkorn University, Bangkok 10330, Thailand; sucharat.tu@student.chula.ac.th; 2 Cell-Based Drug and Health Products Development Research Unit, Chulalongkorn University, Bangkok 10330, Thailand; 3 Department of Molecular Tropical Medicine and Genetics, Faculty of Tropical Medicine, Mahidol University, Bangkok 10400, Thailand; onrapak.rea@mahidol.ac.th; 4 Functional Ingredients and Food Innovation Research Group, National Center for Genetic Engineering and Biotechnology, National Science and Technology Development Agency, Pathumthani 12120, Thailand; sittiruk@biotec.or.th; 5 Research Unit of DNA Barcoding of Thai Medicinal Plants, Chulalongkorn University, Bangkok 10330, Thailand; Suchada.Su@chula.ac.th; 6 Division of Medical Oncology, Department of Medicine, Faculty of Medicine, Chulalongkorn University, Bangkok 10330, Thailand; Chanida.vi@chula.ac.th; 7 Department of Pharmacology and Physiology, Faculty of Pharmaceutical Sciences, Chulalongkorn University, Bangkok 10330, Thailand

**Keywords:** autophagy, polyphenol, stilbene compounds, proteomics, lung cancer, autophagic cell death, apoptosis, AKT/mTOR, Bcl-2

## Abstract

Autophagic cell death (ACD) is an alternative death mechanism in resistant malignant cancer cells. In this study, we demonstrated how polyphenol stilbene compound PE5 exhibits potent ACD-promoting activity in lung cancer cells that may offer an opportunity for novel cancer treatment. Cell death caused by PE5 was found to be concomitant with dramatic autophagy induction, as indicated by acidic vesicle staining, autophagosome, and the LC3 conversion. We further confirmed that the main death induction caused by PE5 was via ACD, since the co-treatment with an autophagy inhibitor could reverse PE5-mediated cell death. Furthermore, the defined mechanism of action and upstream regulatory signals were identified using proteomic analysis. Time-dependent proteomic analysis showed that PE5 affected 2142 and 1996 proteins after 12 and 24 h of treatment, respectively. The crosstalk network comprising 128 proteins that control apoptosis and 25 proteins involved in autophagy was identified. Protein–protein interaction analysis further indicated that the induction of ACD was via AKT/mTOR and Bcl-2 suppression. Western blot analysis confirmed that the active forms of AKT, mTOR, and Bcl-2 were decreased in PE5-treated cells. Taken together, we demonstrated the novel mechanism of PE5 in shifting autophagy toward cell death induction by targeting AKT/mTOR and Bcl-2 suppression.

## 1. Introduction

Lung cancer is the leading cause of morbidity and mortality worldwide [1]. There are several therapies available for lung cancer treatment, including surgery, radiotherapy, and chemotherapy [2]. However, high mortality and low five-year survival rates have been found due to drug resistance [3,4]. Interestingly, previous research revealed that conventional chemotherapy is unable to induce apoptotic cell death in 60% of NSCLC patients, leading to major hurdles to achieving a positive clinical outcome [5]. Therefore, it is important to develop and find new compounds that can induce apoptotic-independent cell death with only a minor incidence of resistance. Despite the role of autophagy as a catabolic process that enhances cell survival through the recycling of bioenergetics and basic cellular units and by the elimination of damaged organelles and unwanted proteins [6,7], several lines of evidence suggest that autophagy is also somehow critical for cell death, especially when the apoptosis mechanism is inhibited or defective [8]. Autophagic cell death (ACD) has also been shown to be important for cell death in cancer cells that are resistant to chemotherapies [6,9,10,11]. Certain drugs or compounds that trigger autophagy are believed to be beneficial for killing cancer cells or for improving the response to conventional drugs [12,13].

Autophagy is induced by phosphatidylinositol 3-kinase (PI3K) type III, which interacts and forms a complex with Atg6 (Beclin1), VPS15, VPS34, and Atg14L [14]. Class III PI3Kpromotes activation of the phagophore [15], which then expands via the functions of the LC3/GABARAP and Atg5–Atg12 pathways, resulting in the formation of autophagosomes. LC3s is important for autophagosome formation, because a cytosolic form of LC3 (LC3-I) is conjugated to phosphatidylethanolamine (PE) to form LC3-II, which is recruited to autophagosomal membranes [16]. Importantly, autophagy is strictly controlled by the protein kinase B (AKT)/mammalian target of rapamycin (mTOR) axis, which is responsible for a variety of cellular signals in nutrient sensing, protein synthesis, survival, and cell growth [17]. The inhibition of mTOR by stress or by the deprivation of nutrients has been evidenced to induce autophagosome formation. The well-known classic mTOR inhibitor rapamycin (Sirolimus) has been widely used in studies aiming at autophagy induction [18,19]. Additionally, rapamycin and other autophagic inducers were shown to exert beneficial effects in a preclinical evaluation for aging and increasing lifespan [20]. Unlike for classical autophagy, the regulatory mechanism of ACD is largely unknown. Recent information has suggested that ACD and apoptosis are interconnected through many nodes of signal crosstalk, leading to coordinated cellular component degradation and cell death [21]. One important central node of crosstalk between apoptosis and autophagy is the Bcl-2 protein. Bcl-2 has been long known to play an important role in the inhibition of apoptosis at the mitochondria [22], and its functions in regulating autophagy are recently becoming clearer. The anti-apoptosis activity of Bcl-2 is based on its ability to antagonize pro-apoptotic members of the Bcl-2 family. Likewise, Beclin 1, a Bcl-2 homology 3 (BH3) domain-only protein [23] that functions as a crucial initiator of autophagosome formation, is known to interact with Bcl-2 [24], and such a protein interaction prevents Beclin 1 from carrying out its function [25]. A previous study demonstrated that the suppression of Bcl-2 by siRNA knockdown could induce ACD but not apoptosis in breast cancer cells [21], so the induction of autophagy together with Bcl-2 suppression could offer a better option for cell death induction for cancer treatment.

Numerous bioactive phytochemicals, especially polyphenols such as stilbenoids and flavonoids, have been discovered in orchids [26,27], including 2-(4″-hydroxybenzyl)-5-2″-dihydroxy-3-methoxystilbene (PE5), which is a natural compound isolated from the roots of *Phragmipedium* sp. [28]. Previous studies have revealed that compounds in stilbenoids, such as resveratrol, have potential anti-cancer effects [29,30,31]. Other stilbenes, such as piceatannol, combretastatin A-4, and pterostilbene, have various pharmacological effects against various type of cancers [31,32,33]. With an aim to investigate the activity of PE5 on ACD induction in lung cancer cells and its regulatory profiles involving the crosstalk between apoptosis and ACD, we utilized molecular pharmacological and proteomic approaches to unravel the full profile of the molecular regulations that could help us gain a better understanding of ACD control in lung cancer as well as the development of a treatment compound for cancer therapy.

## 2. Materials and Methods

### 2.1. Test Compound

Whole plants of *Paphiopedilum exul (Ridl.) Rolfe* were collected from the southern region of Thailand and identified by Associate Professor Thatree Phadungcharoen at the Department of Pharmacognosy and Pharmaceutical Botany, Chulalongkorn University, Thailand. The fresh roots were separated from the plants, cleaned, and dried at a temperature of 50 °C. Voucher specimens (RS15011) were deposited at the Herbarium of Natural Medicine, Chulalongkorn University, Thailand. The dried roots of *P. exul* (380 g) were powdered and macerated three times with methanol at room temperature. The methanol extract was concentrated under reduced pressure and combined to yield 100 g of crude methanol extract.

The methanol extract (50 g) was separated on a silica gel column (1.25 kg, 10 × 40 cm) and washed down with n-hexane/acetone (3:1) to yield eight fractions (A–H). These fractions were assayed by cytotoxicity activity. Fraction E (9.9 g), the most active fraction, was subjected to further separation. Fraction E (9.9 g), was separated on a silica gel column (300 g, 4.5 × 40 cm) and washed down with n-hexane/acetone (2:1) to provide six subfractions (E1–E6). Size exclusion chromatography of fraction E5 (780 mg) on a Sephadex LH-20 column eluted with methanol yielded four subfractions (E51-E54). Separation of subfraction E53 (290 mg) using a silica gel column (15 g, 2 × 14 cm), eluted with CH_2_Cl_2_/acetone (20:1), gave five subfractions (E531–E535). PE5 (58.0 mg) was obtained from subfraction E534. The purity of PE5 was shown in Supplement Data. PE5 was be dissolved in DMSO and media to achieve the desired concentrations, containing less than 0.5% DMSO at final dilution.

### 2.2. Cell Culture

Non-small cell lung cancer cells used in the experiments including NCI-H460 [H460] (ATCC^®^ HTB-177™, RRID: CVCL_0459), NCI-H292 [H292] (ATCC^®^ CRL-1848™, RRID: CVCL_0455), A549 (ATCC^®^ CCL-185™, RRID: CVCL_0023), and BEAS-2B (ATCC^®^ CRL-9609™, a normal human bronchial epithelium) and HCT116 (ATCC^®^ CCL-247™, a human colorectal carcinoma cell line) were obtained from the American Type Culture Collection (Manassas, VA, USA). Primary dermal papilla (DP) was purchased from Celprogen (Benelux, The Netherlands). H460, H292, and HCT116 were cultured in RPMI (Roswell Park Memorial Institute) 1640, whereas BEAS-2B, A549, and DP were cultured in DMEM (Dulbecco’s Modified Eagle Medium) supplemented with 10% fetal bovine serum (FBS), 100 units/mL penicillin/streptomycin and 2 mM L-glutamine. The cells were incubated in a 5% CO_2_ environment at 37 °C. The cells that reached 70–80% confluence were used for the next experiments.

### 2.3. Chemicals

Cis-diamminedichloroplatinum II (cisplatin, CDDP, 3-(4,5-dimethylthiazol-2-yl)-2,5-diphenyltetrazolium bromide (MTT), Z-VAD-FMK, and Mono-Dansylcadaverine were obtained from Sigma Chemical, Inc. (St. Louis, MO, USA). Hoechst 33342 was obtained from Molecular Probes, Inc. (Eugene, OR, USA). Antibodies for AKT, p-AKT (Ser473), p-mTOR (Ser2448), mTOR, LC3B, PARP, caspase3, ATG5, ATG7, p62, Bcl-2, Bax, and GAPDH, as well as peroxidase-conjugated secondary antibodies, were obtained from Cell Signaling Technology, Inc. (Danvers, MA, USA). RIPA buffer, rapamycin, and wortmannin were also obtained from Cell Signaling Technology, Inc. (Danvers, MA, USA).

### 2.4. Cytotoxicity Assays

All cells were seeded in 96-well plates at the density of 1 × 10^5^ cells/well. After incubating overnight, they were treated with different concentrations of PE5 (0–100 µM) for 24–48 h. Then, 100 μL per well of MTT solution was added to achieve a final concentration of 400 μg/mL and incubated for an additional 3 h at 37 °C. The yellow supernatants of MTT were removed and replaced with 100 μL of Dimethyl sulfoxide (DMSO) to dissolve formazan crystals. The quantity of formazan product proportional to the number of viable cells was measured by recording transformation in absorbance at 570 nm by a microplate reader (Anthros, Durham, NC, USA). The percentage of cell viability and IC50 were determined as described in the manufacturer’s protocol (7sea Biotech, Shanghai, China). Cell viability = (ODexperiment − ODblank)/(ODcontrol − ODblank) × 100%.

### 2.5. Cell Death Assay

The apoptosis cells were analyzed using a fluorescent DNA staining assay with Hoechst 33342 (Molecular Probes, Eugene, OR, USA). The cells were seeded in 96-well plates for 24 h. The cells were treated with PE5 at concentrations of 0–100 µM for 24 and 48 h. Then, cells were incubated with 10 µg/mL Hoechst 33342 for 15 min. Then, cells were visualized using fluorescence microscopy (Olympus IX51 with DP70, Melville, NY, USA).

### 2.6. Flow Cytometry for Apoptosis

Cells in 96-well plates were treated with PE5 at concentrations 50 and 100 µM for 24 h. Then, treated cells were washed with PBS and then were re-suspended in a binding buffer, followed by 20 μg/mL Annexin V-FITC (KeyGENE BioTECH, Nanjing, China) staining and 20 μg/mL propidium iodide (PI, Thermo Fisher ScientificThermo Fisher Scientific, Waltham, MA, USA) incubation for 15 min at the room temperature, based on the manufacturer’s instruction. Finally, 200 μL binding buffer was added into cells, and flow cytometry (BD Bioscience, San Francisco, CA, USA) was used to assess the cells.

### 2.7. Western Blot Analysis

Cells were seeded at density 3 × 10^5^ in a six-well plate for 24 h. The cells were treated with PE5 at concentrations 0–100 µM for 24 h (concentration dependent) and were treated with PE5 at 50 µM for 6, 12, 24, and 48 h (time dependent). Then, cells were incubated on ice for 40 min with RIPA buffer, 1% Triton X-100, 100 mM PMSF, and a protease inhibitor. Cell lysates were analyzed for protein content using the BCA protein assay kit from Pierce Biotechnology (Rockford, IL, USA). Equal amounts of denatured protein samples (60 µg) were loaded onto 10–15% SDS-PAGE for various proteins. Then, the gels were transferred to 0.2 µm PVDF membranes (Bio-Rad, Hercules, CA, USA). The transferred membranes were blocked with medium (25 mM Tris-HCl (pH 7.5), 125 mM NaCl, and 0.05% Tween20 (TBST)) containing 5% non-fat dry milk powder for 1 h, and were incubated overnight with specific primary antibodies. After the overnight block, the membranes were washed three times with TBST and were incubated with the following appropriate horseradish peroxidase-labelled secondary antibodies: anti-rabbit IgG or anti-mouse, for 2 h at room temperature. The immune complexes were detected by SuperSignalWest Pico chemiluminescent substrate (Pierce Biotechnology, Rockford, IL, USA) and were exposed to the film.

### 2.8. Transmission Electron Microscopy

Cultured H460 cells were treated with PE5 at concentration of 50 µM for 24 h. Cells were harvested, washed with PBS, and then fixed in 2.5% glutaraldehyde for 4 h at 4 °C. The samples were washed with PBS and then were treated with 1% osmium tetroxide for 1 h. After the samples had been washed, they were dehydrated in a graded series of ethanol (50%, 70%, and 90%) and acetone and then were embedded in durcupan resin. Thin sections (120 μM) were post-stained with uranyl acetate and lead citrate before examination under JEM-1011 transmission electronic microscope (TEM) (Tokyo, Japan).

### 2.9. Monodansylcadaverine Staining

Cultured H460 cells were treated with PE5 at concentration of 50 µM for 24 h in 96 well plates. After 24 h, cells were stained with MDC (50 μmol/L) for 30 min at 37 °C and then cells were visualized using fluorescence microscopy (Olympus IX51 with DP70, Melville, NY, USA).

### 2.10. Immunofluorescence

H460 cells were seeded onto coverslips in a six-well plate and incubated overnight. After the treatment, the cells on coverslips were fixed with 4% paraformaldehyde for 30 min and permeabilized with 0.1% Triton-X for 20 min. Then, the cells were incubated with 3% bovine serum albumin (BSA) for 30 min to prevent nonspecific binding. Next, the cells were washed and incubated with rabbit anti-LC3B antibody for 1 h at room temperature. Primary antibody was removed, and the cells were washed and subsequently incubated with Alexa Fluor 488 (Invitrogen, Waltham, MA, USA) conjugated goat anti-rabbit IgG (H + L) secondary antibody for 1 h at room temperature. Samples were washed with PBS, then visualized and imaged by fluorescence microscope (Olympus IX 51 with DP70, Olympus America Inc., Center Valley, PA, USA).

### 2.11. Mass Spectrometry-Based Proteomics

H460 cells were seeded at a density of 1 × 10^6^ cells in a 10 cm^2^ dish for 24 h. Then, the cells were treated with PE5 at concentration of 50 µM for 12 and 24 h. After being treated, cells were washed with PBS and incubated with lysis buffer (1% SDS, 1% NaCl, 1% triton-X and protease inhibitor). The lysates were disrupted by an ultrasound sonicator (Sonics & Materials, Newtown, CT, USA) on ice for 5 s × 10 with an interval of 2 min. The protein lysates were centrifuged at 12,000× *g* for 5 min at 4 °C to remove cell debris. Cell lysates were analyzed for protein content using Quick Start™ Bradford Protein Assay (Bio-Rad, Hercules, CA, USA). The protein samples (30 µg) were loaded onto 12% SDS-PAGE. To detect the protein bands, the SDS-polyacrylamide gels were stained with Coomassie blue G-250 (Bio-Rad, Hercules, CA, USA). After that, the gel was de-stained, until clear bands were visible. Each gel lane was cut into small pieces and were kept at −20 °C until use. Gel pieces were de-stained by 50% acetonitrile in 50 mM ammonium bicarbonate. The colorless gel pieces were reduced and alkylated by dithiothreitol and iodoacetamide, then dehydrated by acetonitrile, and were allowed to dry completely in a fume hood. To rehydrate the gel pieces, trypsin solution at a concentration of 0.02 mg/mL in 50 mM ammonium bicarbonate (Sigma, Saint Louis, MO, USA) was added to the tubes, and each gel piece was incubated at 37 °C overnight. Peptides were extracted by addition of acetonitrile, and the solution was shaken for 15 min. The supernatant was collected, and the peptide mixtures were completely dried by a speed-vac (Eppendorf, Hamburg, Germany). The samples were resuspended in 0.1% formic acid and subjected to an Ultimate^®^ 3000 Nano-LC system analysis (Thermo Scientific, Waltham, MA, USA) coupled with a microTOF-Q II (Bruker, Bremen, Germany). The mass spectrometric data was converted to .mgf file using DataAnalysis™ software version 3.4 (Bruker, Germany). Mascot server (Matrix Science, Boston, MA, USA) against human NCBI database. The significance threshold for protein identification was set at 95%. Three biological replications were performed. The gene ontology classification and protein-protein interaction were performed using Panther and STITCH software, respectively.

### 2.12. Small Interfering RNA (siRNA) Transfection

H460 cells were seeded into a 24-well plate and allowed to reach approximately 70% confluence on the day of transfection. The siRNA including siRNA control and siRNA against ATG7 were obtained from Cell Signaling Technology, Inc. (Danvers, MA, USA). Cells were transfected with 100 nM siRNA using Lipofectamine 2000 (Invitrogen), according to the manufacturer’s instructions [10].

### 2.13. Patient-Derived Primary Lung Cancer Cell Line from Malignant Pleural Effusion

The patient-derived malignant cancer cells were isolated from pleural effusions of advanced stage NSCLC patients who had been diagnosed at the King Chulalongkorn Memorial Hospital. The protocol of conduction was approved by the Ethics Committee of the Faculty of Medicine, Chulalongkorn University, Bangkok, Thailand (IRB 365/62), and was obtained informed consents from all participants. ELC08 and ELC10 were collected from pleural effusion (1000 mL) through thoracentesis. After culturing for 10 passages, they were characterized as the patient-derived primary cancer cell lines. This study was carried out in accordance with the principles of World Medical Association Declaration of Helsinki.

### 2.14. Statistical Analysis

Data was given as the means ± standard deviation of at least three independent replicated samples procedures composed of at least three independent replicated samples. Multiple comparisons for statistically significant differences between multiple groups (ANOVA) were calculated by using SPSS software program version 16 (SPSS Inc., Chicago, IL, USA), followed by individual comparisons with Scheffe’s post-hoc test. For two-group comparisons, t-test was calculated by using the program same as previous calculation. Differences were be considered statistically significant for (*) *p* < 0.05 and (**) *p* < 0.01. GraphPad prism 5 was used for creating graphs in all experiments (GraphPad Software, San Diego, CA, USA).

## 3. Results

### 3.1. Anti-Cancer Activities of PE5 on Lung Cancer Cells

**Figure 1:**
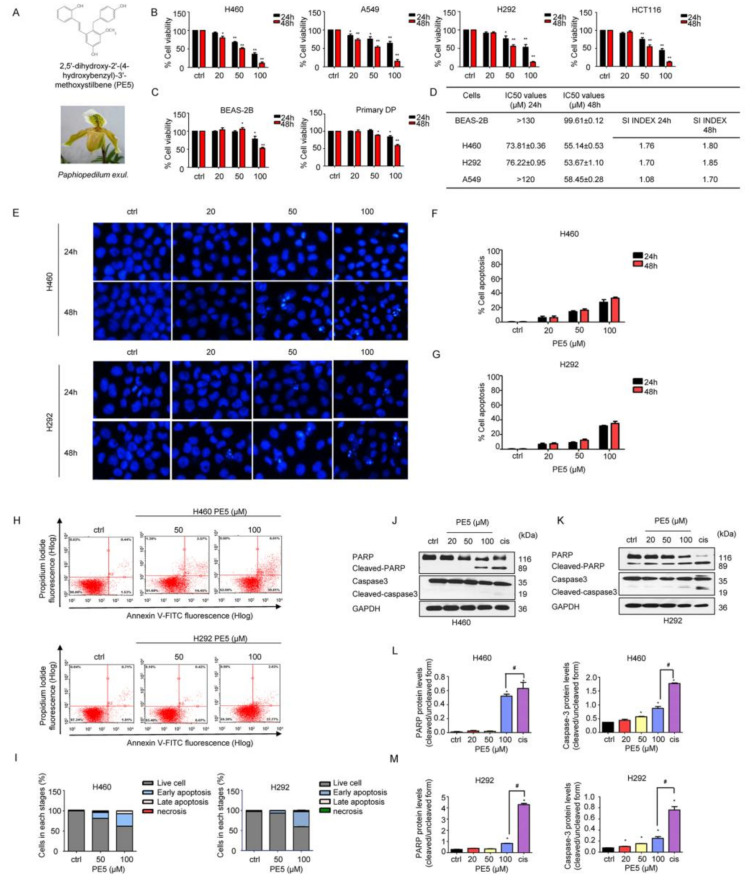
Effects of PE5 on cell viability and apoptotic cell death in non-small cell lung cancer. (**A**) PE5 structure and the plant specimen of *Paphiopedilum exul.* (**B**) All cells were treated for 24–48 h and analyzed by MTT assay. Graphs showing the percentages of cell viability. (**C**) Normal cells were similarly treated for 24–48 h and analyzed for the percentages of cell viability. (**D**) The IC50 and selectivity index (SI) in all cells were calculated for each cell type. (**E**–**G**) Cells were seeded and treated for 24 h before adding Hoechst 33342 to stain the cell nucleuses. Images were detected by using a fluorescence microscope, and the percentages of nuclear-fragmented were calculated. (**H**–**I**) Apoptotic and necrotic cells were determined using annexin V-FITC/PI staining with flow cytometry. (**J**–**M**) Apoptosis-related proteins were measured by western blot analysis. Cells were treated and were detected caspase3, PARP, cleaved caspase3, and cleaved PARP protein levels. The blots were reprobed with GAPDH to confirm equal loading of the protein samples. The relative protein levels were calculated by densitometry. Data represent the mean ± SD (n = 3), (* *p* < 0.05, ** *p* < 0.01, compared with the untreated control), and (^#^ *p* < 0.05, compared with cisplatin).

To elucidate the anti-cancer potential of PE5 (Figure 1A), we first determined the cytotoxic profile of PE5 in several non-small cell lung cancer (NSCLC) cells, including the H460, H292, and A549 cell lines. In addition, we also determined the cytotoxic effect of the compound in other cell lines, including the colon cancer cell line (HCT116), primary dermal papilla (DP), and human bronchial epithelial cells (BEAS-2B), for comparison. Cell viability was evaluated by the MTT assay. Cells were incubated with various concentrations of PE5 (0–100 µM) for 24–48 h. The results showed that PE5 significantly reduced cell viability in all NSCLCs and colon cancer cells (Figure 1B), while it had a slightly toxic effect on non-cancerous DP and BEAS-2B cells (Figure 1C). In addition, we also calculated the selectivity index (SI), which represents the IC50 value for the normal cell line/IC50 value for the cancerous cell line after 24–48 h of PE5 treatment. The results showed that the SI index for H460 and H292 cells was higher than that for A549 cells (Figure 1D).

Apoptosis is an effective program that eliminates undesirable cells, senescent cells, and genetically damaged cells [34]. It is characterized by blebbing of the cell membrane, cell shrinkage, and the condensation of DNA. Therefore, we examined whether the cytotoxic effect of PE5 was involved in apoptosis. Hoechst 33342 staining was used to evaluate the nucleus morphology of the PE5-treated cells. The NSCLC cells were treated with PE5 at the concentrations of 0–100 µM for 24–48 h. The results revealed that PE5 caused apoptosis with DNA condensation and/or fragmentation in H460 and H292 cells (Figure 1E–G). To confirm this, flow cytometric analysis of apoptosis using annexin V-FITC/PI staining was utilized. The results showed that PE5 induced apoptosis in a concentration-dependent manner (Figure 1H–I). As shown in Figure 1H, the percentages of apoptotic cells in response to 50 and 100 µM of PE5 were 17.02% and 37.42%, respectively, in H460 cells. In addition, the percentages of apoptotic cells in response to 50 and 100 µM of PE5 in H292 cells were 6.49% and 39.80%, respectively. The apoptotic protein markers, including caspase-3, poly (ADP-ribose) polymerase (PARP), and their cleaved forms, were determined by western blot analysis. We treated H460 and H292 cells with various concentrations of PE5 for 24 h and used cisplatin, a standard treatment drug for lung cancer that is known for apoptosis induction, as an internal control (at its IC50 concentration). Western blotting revealed that, in response to PE5 treatment, the activated caspase-3 was significantly increased compared with the untreated control. Likewise, the cleaved form of PARP was upregulated simultaneously (Figure 1J–M). In addition, the activated caspase-3 and the cleaved form of PARP when treated with cisplatin were significantly increased compared with treatment with 100 µM of PE5 in all the NSCLC cell lines.

### 3.2. PE5-Induced Autophagy in Lung Cancer Cells

**Figure 2:**
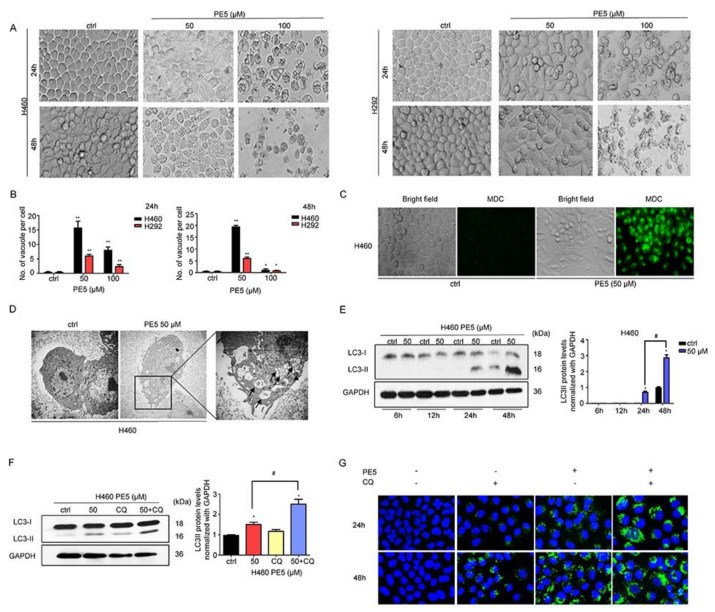
Effect of PE5 on autophagy induction and autophagic flux in lung cancer cells. (**A**,**B**) the morphological changes of the cells were detected using a microscope and the number of vacuoles per cells were calculated after H460 and H292 cells were treated with PE5. (**C**) H460 cells were treated with PE5 and stained with monodansylcadaverine (50 μmol/L) and visualized by fluorescence microscopy (Olympus IX51 with DP70). (**D**) H460 cells were treated with 50 μM PE5 for 24 h and observed by transmission electron microscopy. Arrowheads indicate the autophagosomes and the arrows show the vacuoles. (**E**) After getting treated with indicated amounts of PE5 for indicated time periods (6–48 h), LC3 proteins were measured by western blot analysis. The blots were reprobed with GAPDH to confirm equal loading of the protein samples. (**F**,**G**) Autophagy flux was determined in 50 μM PE5-treated cells in the presence of chloroquine (10 µM). The LC3 proteins were determined by western blot analysis. The blots were reprobed with GAPDH to confirm equal loading of the protein samples. The cells were treated 50 μM PE5 with or without chloroquine (10 µM) for 24–48 h. The level of LC3 expression was analyzed by immunofluorescence staining. Data represent the mean ± SD (n = 3) and (* *p* < 0.05, ** *p* < 0.01, compared with the untreated control) (^#^ *p* < 0.05, compared with PE5-treated alone or different time).

Autophagy has been shown to mediate cell death in malignant cells and has been proposed as a possible means of achieving cancer elimination [6]. We next observed NSCLC cells after treatment with various concentrations of PE5 for 24–48 h and found that cytoplasmic vacuoles were clearly noticeable after treatment with 50–100 μM of PE5 in a time- and concentration-dependent manner (Figure 2A,B). In the cells treated with 100 μM PE5, cytoplasmic contraction, a morphological feature of typical apoptosis, was observed, and most of the cells were detached at 48 h. Next, we treated the cells with PE5 (50 µM) for 24 h and stained them for autolysosome using monodansylcadaverine (50 μmol/L). The results indicated that the vacuoles observed in the PE5-treated cells were mostly stained with the monodansylcadaverine compared with those of the non-treated control cells (Figure 2C). Furthermore, the cytoplasmic vacuoles were observed by transmission electron microscopy (TEM) (Figure 2D). In the PE5-treated cells, we found degraded cell materials inside the double membrane vesicles, which appeared to be autophagosomes (Figure 2D, indicated with arrowhead). The vacuoles in which all their contents were degraded (empty vesicles) were likely to be autophagosomes fused with lysosomes, representing active autolysosomes (Figure 2D, indicated with arrows). Western blot analysis was carried out to confirm the expression of autophagy-related marker proteins, such as LC3-I to II conversion. The protein analysis further confirmed autophagy induction in the PE5-treated cells, as the increasing of LC3-II could be clearly observed in a time-dependent manner after PE5 treatment (Figure 2E).

It should be noted that the increased formation of autophagosomes or LC3 conversion may not directly indicate autophagic flux. Therefore, we measured the level of LC3-II in the presence of 10 μM chloroquine (CQ). When autophagosome–lysosome fusion was inhibited by the activity of CQ in PE5-treated cells, the level of LC3II significantly increased compared to PE5 treated control (Figure 2F). In addition, we confirmed the results by immunofluorescence detecting punctate LC3 in PE5-treated cells in the presence of CQ over a time course. The results showed that CQ increased the appearance of punctate LC3 staining (Figure 2G).

**Figure 3:**
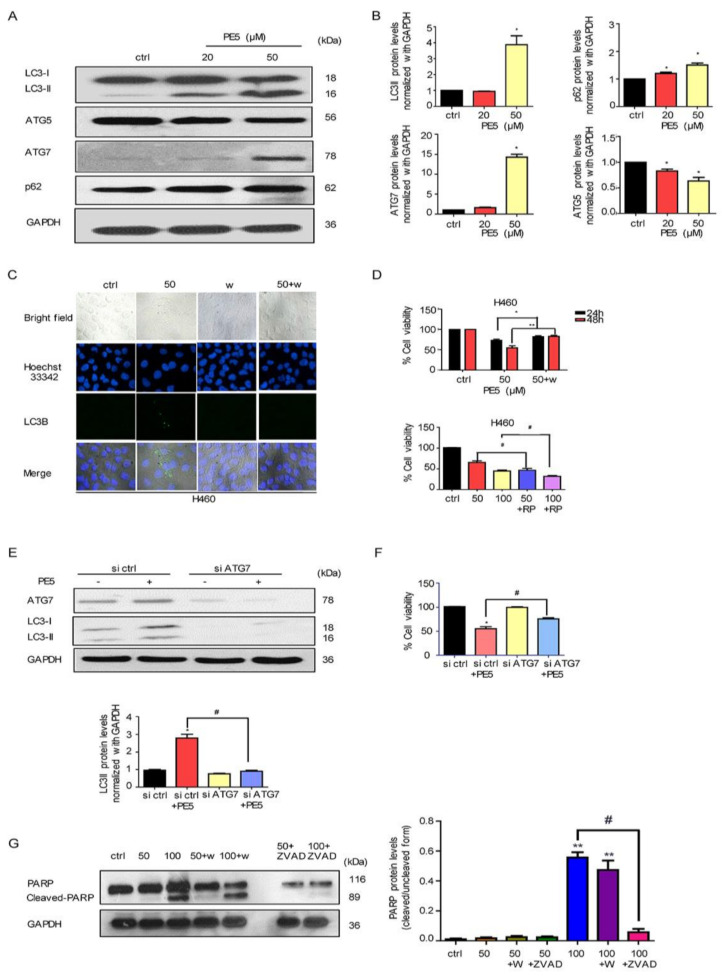
Effect of PE5 on autophagy regulatory proteins and autophagic cell death. (**A**,**B**) H460 cells were treated with PE5 and detected for LC3, p62, ATG5, and ATG7 proteins by western blotting. The blots were reprobed with GAPDH to confirm equal loading of the protein samples. (**C**) H460 cells were pre-treated with wortmannin (1 µM) (an autophagic inhibitor) and treated with PE5 for 24 h. Expression of LC3 was analyzed by immunofluorescence staining. (**D**) H460 cells were treated with PE5 in the presence of wortmannin (1 µM) or rapamycin (200 nM). Cell viability was analyzed by MTT assay. (**E**,**F**) Cells were transfected with siATG7 and treated with 50 µM PE5 for 24 h. Expression levels of each ATG and LC3 were assessed by western blot analysis. The blots were reprobed with GAPDH to confirm equal loading of the protein samples. Cell viability was assessed using the MTT assay at 48 h. (**G**) H460 cells were pre-treated with wortmannin (1 µM) (an autophagic inhibitor) or Z-VAD-FMK (20 μM) (apoptosis inhibitor) and treated with PE5 for 24 h. Expression of PARP and cleaved PARP were analyzed by western blot analysis. The blots were reprobed with GAPDH to confirm equal loading of the protein samples. Data represent the mean ± SD (n = 3) and (* *p* < 0.05, ** *p* < 0.01, compared with the untreated control) (^#^ *p* < 0.05, compared with PE5-treated alone and significantly different from siATG-transfected cells).

In the autophagy process, LC3, SQSTM1/p62, and many ATG proteins are recruited to phagophores for inducing the phagophores to undergo an expansion step. Therefore, we determined the expression of LC3, ATG5, ATG7, and p62 by western blot analysis. In response to PE5 treatment, the protein analysis showed the sufficient conversion of LC3-I to LC3-II along with the increase in p62 and ATG7; however, ATG5 was found to have slightly decreased (Figure 3A,B).

Having shown the cell killing as well as the potent autophagic-induction activities of the compounds, the next question was whether the autophagy induced by PE5 in our system was associated with a cytotoxic action. Consequently, we co-treated the cells with PE5 and wortmannin (an autophagy inhibitor) and analyzed the autophagosomes and cell viability. Figure 3C shows that the addition of the autophagy inhibitor completely abolished the formation of autophagosomes induced by PE5, as indicated by the absence of LC3 puncta in the autophagosomes in comparison to those of the PE5-treated cells. Figure 3D shows that wortmannin could reverse the cytotoxic effect induced by PE5. In contrast, rapamycin (autophagy inducer) significantly enhanced cytotoxic effect of PE5. To confirm, the precise suppression of autophagy was conducted using the siRNA approach. Results indicated that siATG7 transfection caused a dramatic depletion of ATG7 and effectively attenuated the PE5-induced conversion of LC3-I to II, as shown in Figure 3E. Importantly, siATG7 significantly decreased PE5-induced cell death (Figure 3F).

In addition, we utilized apoptosis and autophagy inhibitors to test the involvement of autophagy on apoptosis response. The cells were treated with PE5 in the presence of Z-VAD-FMK (caspase inhibitor) or wortmannin (autophagy inhibitor) and analyzed for cleaved-PARP. The results showed that the cleaved form of PARP was increased in response to PE5 and ZVAD could inhibit the cleaved-PARP. In contrast, the autophagy inhibitor had no significant effect on cleaved-PARP in PE5-treaded cells (Figure 3G).

### 3.3. Molecular Functions and Biological Processes of the Proteins in PE5-Treated Cells

**Figure 4:**
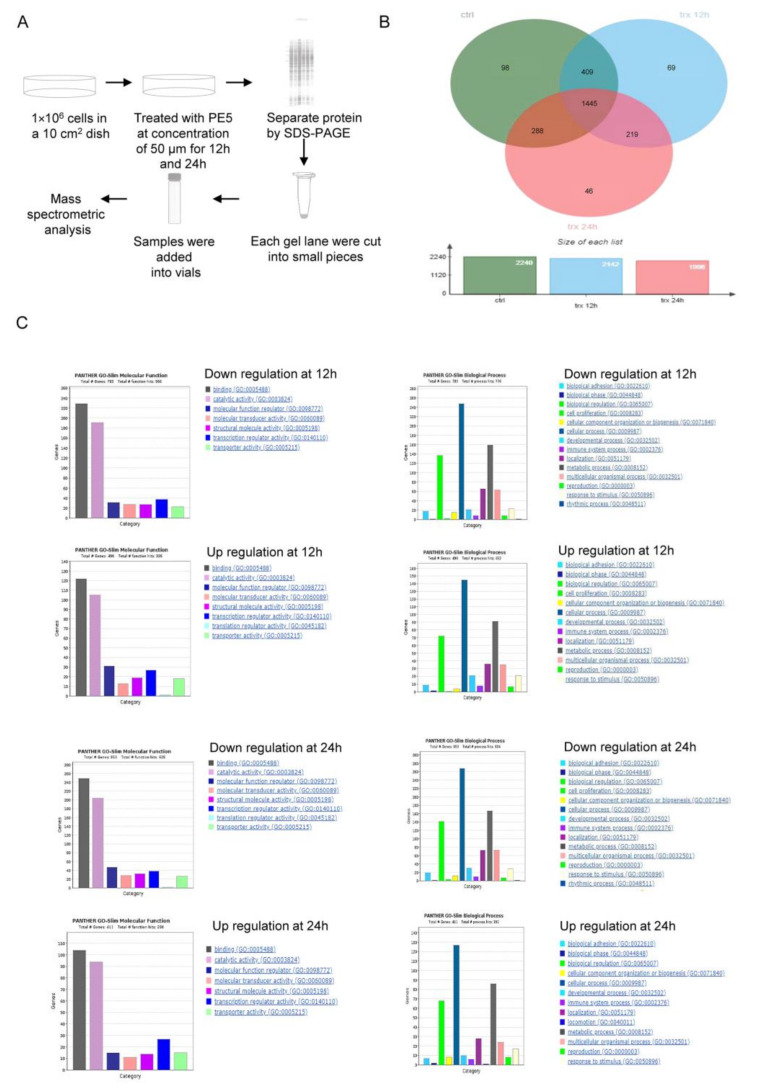
Proteomic analysis of PE5-treated cells. (**A**) Brief method involving proteomic analysis and mass spectrometric analysis. (**B**) Venn diagram (analyzed by jVenn software; http://jvenn.toulouse.inra.fr/app/example.html, access date: 1 January 2020) showing the different proteins between the control and PE5-treated cells at 12 and 24 h. (**C**) Gene ontology classification according to the biological process and molecular function terms of the upregulated and downregulated proteins using Panther software (Panther software; http://www.pantherdb.org/, access date: 20 January 2020).

It is essential to determine the full profile of the proteins and signals affected by a pharmacological compound in order to define its major mechanism of action. Proteomic studies have generated numerous datasets of therapeutic significance in cancer [35]. In this study, we used proteomics to identify the proteins that control ACD as induced by PE5. Here, cells were seeded in a 10 cm^2^ dish and treated with PE5 at a concentration of 50 µM for 12 and 24 h. Next, we separated the proteins by SDS-PAGE and analyzed the proteins using mass spectrometric analysis (Figure 4A). In our setting, the proteins affected by the treatment were determined at two different time points (12 and 24 h) for determining the time-dependent mechanisms and for defining the crosstalk between apoptosis and ACD. The total number of proteins identified from the control non-treated cells was 2240 proteins, while from the PE5-treated cells there were 2142 and 1998 proteins at 12 and 24 h, respectively. The protein lists from the control and treated cells were input to a Venn diagram. The results showed that 69 proteins were uniquely found in the PE5-treated cells at 12 h, and 46 proteins were specifically found in the PE5-treated cells at 24 h (Figure 4B). Furthermore, the proteins that were significantly altered at 12 and 24 h were subjected to further molecular function and biological process analysis using Panther software (Figure 4C).

**Figure 5:**
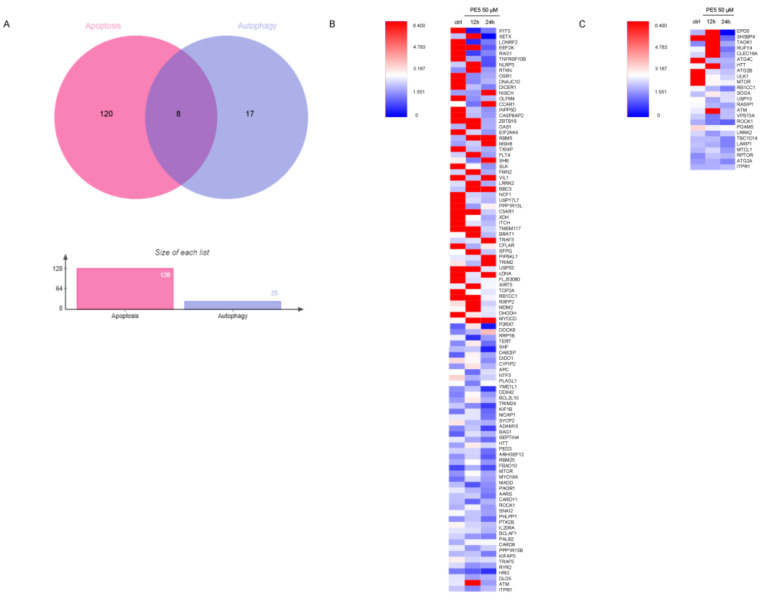
Protein alteration in apoptosis and autophagy pathway. (**A**) Venn diagram was analyzed by jVenn software; http://jvenn.toulouse.inra.fr/app/example.html, access date: 16 June 2020. This diagram represented the number of different apoptosis and autophagy proteins affected by PE5. (**B**) Heatmap represented the levels of 128 proteins in regulating apoptosis signaling pathways in the control and 50 μM PE5 groups at 12 and 24 h using the MultiExperiment Viewer (MeV) in the TM4 suite software (http://mev.tm4.org/#/welcome, access date: 22 June 2020). (**C**) Heatmap represented the levels of 25 proteins in regulating autophagy pathway in the control and 50 μM PE5 groups at 12 and 24 h using the MultiExperiment Viewer (MeV) in the TM4 suite software (http://mev.tm4.org/#/welcome, access date: 22 June 2020).

From gene ontology (biological process) and gene ontology (molecular function) analysis, our proteomic analysis evidenced that PE5 directly or indirectly modulated the expression of proteins that regulate apoptosis and the autophagy processes (Figure 5A). We identified that PE5 affected 128 apoptosis-related proteins as well as 25 proteins of autophagy regulation. In addition, eight proteins altered by PE5 were found to be involved in both apoptosis and autophagy.

As shown in Figure 5, the proteins involved in apoptosis process (Figure 5B) and the autophagy proteins (Figure 5C) were altered in a time-dependent manner. It was noteworthy that the apoptosis proteins were upregulated at 12 h with some of them, such as NISCH, CCAR1, RBM5, MSH6, BBC3, TRAF3, PIP5KL1, and MYOCD, maintained at a high level until 24 h, while most of the autophagy proteins were upregulated at 12 h but then dropped afterward. For autophagy, the level of the mTOR protein was found to be critically decreased in a time-dependent manner. The proteins involved in the apoptosis pathway, such as INPP5D, CASP8AP2, EEF2K, DICER1, EIF2AK4, TXNIP, SLK, NCF1, USP17L7, C5AR1, XDH, ITCH, TMEM117, USP53, TOP2A, RB1CC1, DHODH, DIDO1, and TNFRSF10B, were significantly downregulated in a time-dependent manner (Figure 5B).

### 3.4. Mechanisms of Action of PE5 Analyzed by the Protein–Protein Interaction Networks and Signaling Pathways

**Figure 6:**
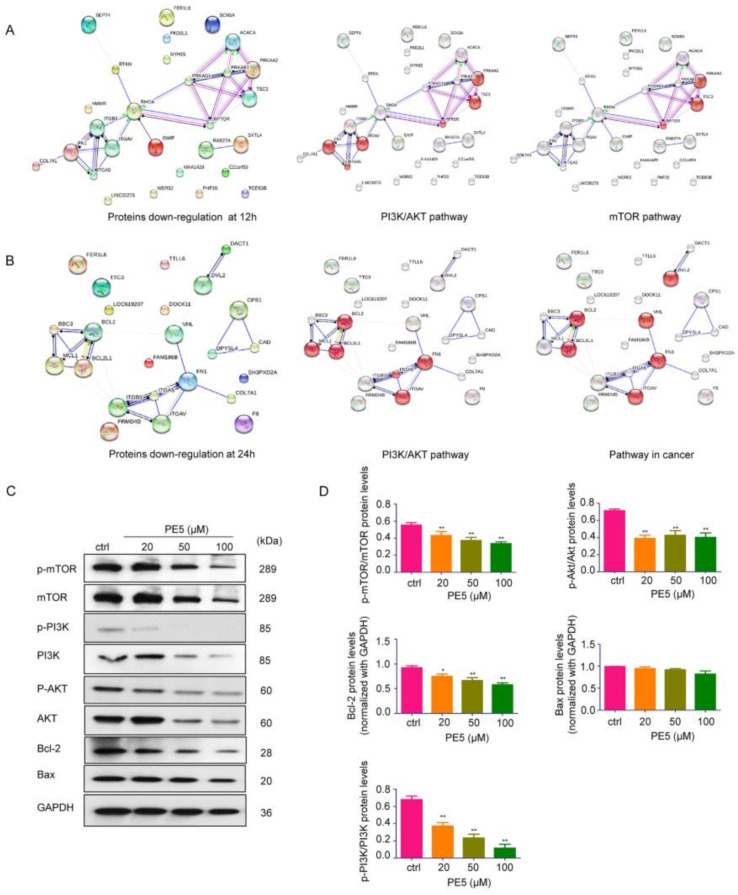
Networks of functional protein–protein interactions of the top 20 downregulated proteins in response to PE5. (**A**,**B**) The functional protein–protein interactions of the top 20 downregulated proteins, and the significant nodes of each network were identified and rebuilt as a network of the signaling pathway in cancer using STITCH database version 5.0 (http://stitch.embl.de/, access date: 24 June 2020). (**C**,**D**) The key proteins p-PI3K, PI3K, mTOR, p-mTOR, AKT, p-AKT, Bcl-2, and Bax were determined by western blotting in H460 cells, and the immunoblot signal intensities were quantified by densitometry. Data represent the mean ± SD (n = 3), (* *p* < 0.05, ** *p* < 0.01, compared with the untreated control).

The hyperactivity or overexpression of some groups of proteins, such as the PI3K/AKT/mTOR and Bcl-2 family proteins, can be found in lung cancer [36]. In order to identify the major contributing mechanism of action of PE5, the proteins in PE5-treated cells at different times were subjected to protein–protein interaction network analysis with the STITCH database and with interactions contained in the STRING database in order to determine the significant kinase pathways. The resulting networks at 12 and 24 h are presented in Figure 6A,B, respectively. The top 20 most downregulated proteins were subjected to STITCH for network node evaluation and for the prediction of their molecular function. The results revealed that proteins such as RTKN and PRKAA2 interacted with PI3K/AKT and the mTOR pathway at 12 h (Figure 6A). In addition, the BBC3 protein was found to link with the anti-apoptotic Bcl-2 and Mcl-1 proteins. Regarding the proteins involving malignant phenotypes, the FN1 protein was found to be associated with PI3K/AKT and its pathway in cancer (Figure 6B). These results suggested that the major mechanisms by which PE5 mediated ACD was through the potent and sustained suppression of the PI3K/AKT/mTOR axis as well as by the depletion of Bcl-2 at 24 h.

Having shown the node of protein interaction as well as the dominant mechanisms of PE5, we further confirmed the significance of the proteomics finding with western blot protein analysis using specific phosphorylated antibodies against the active forms of PI3K, AKT, and mTOR proteins. In addition, we validated the effect of the compound on Bcl-2. The results indicated that treatment of the cells with PE5 significantly decreased the level of p-PI3K, p-AKT, and p-mTOR proteins in comparison to that of the non-treated control. Moreover, PE5 significantly decreased the level of Bcl-2 proteins (Figure 6C,D).

### 3.5. Effect of PE5 on Patient-Derived Primary Lung Cancer Cells

**Figure 7:**
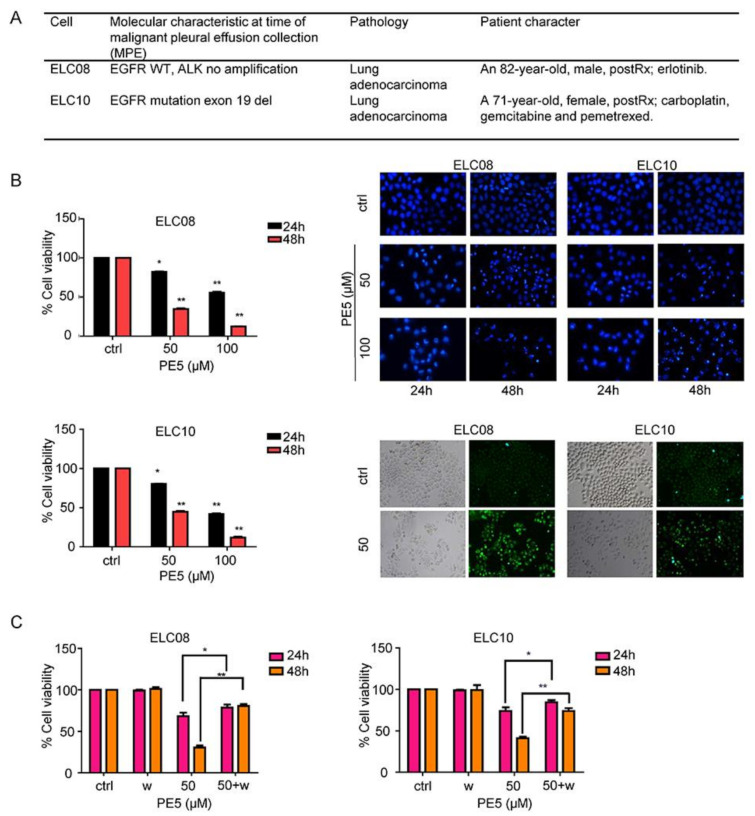
Effect of PE5 on patient-derived primary lung cancer cells. (**A**) The morphology of patient-derived primary cancer cell lines (ELC08 and ELC10) and their molecular characteristics. (**B**) Graphs showing the percentages of cell viability. All cells were treated for 24–48 h and analyzed by MTT assay. Apoptotic nuclei in the cells treated with PE5, determined by Hoechst 33342 staining and visualized by fluorescence microscopy. Cells were treated with PE5 and stained with monodansylcadaverine (50 μmol/L) and visualized by fluorescence microscopy (Olympus IX51 with DP70). (**C**) H460 cells were treated with PE5 in the presence of wortmannin (1 µM). Cell viability was analyzed by MTT assay. Data represent the mean ± SD (n = 3), (* *p* < 0.05, ** *p* < 0.01, compared with the untreated control).

Chemotherapeutic drug resistance is recognized as a major cause of therapeutic failure and tumor recurrence in lung cancer. Autophagy is a cellular process responsible for cell survival, but in some apoptosis-resistant cancer cells, autophagy can also increase anti-cancer effect through autophagy-mediated mechanisms of cell death [8]. To characterize the potency of the anti-cancer activity of PE5, we determined the cytotoxic profile of PE5 in chemotherapeutic resistant primary lung cancer cells (ELC08 and ELC10) and induction of autophagy by using MTT assay, Hoechst33342, and MDC staining, respectively, as shown in Figure 7. The basic cell morphology of the patient-derived primary cancer cell lines and the molecular characteristics are shown in Figure 7A. The results indicated that PE5 exerted a cytotoxic potency against chemotherapeutic resistant primary lung cancer cells (Figure 7B). The apoptotic cell death was further evaluated by Hoechst33342 staining. The results revealed that PE5 caused slightly increase in apoptosis in a concentration- and time-dependent manner. To confirm the autophagy characteristic, we treated the cells with PE5 and stained them for autolysosome using monodansylcadaverine (50 μmol/L). The results indicated that the vacuoles observed in the PE5-treated cells were mostly stained with the monodansylcadaverine compared with those of the non-treated control cells. Next, we determined whether the autophagy induced by PE5 was associated with a cytotoxic action in primary lung cancer cells. We co-treated primary lung cancer cells with PE5 and wortmannin and analyzed for cell viability. Figure 7C showed that the autophagy inhibitor could reverse the cytotoxic effect induced by PE5 in primary lung cancer cells. Therefore, autophagy process can involve in cell death in resistant primary lung cancer cells caused by PE5.

## 4. Discussion

The incidence of lung cancer is continuing to increase worldwide. At present, platinum-based drugs, such as cisplatin and paclitaxel, are commonly used for the treatment of lung cancer patients in the clinic [37]. Unfortunately, it is common for these patients to develop drug resistance. Resistance to platinum-based drugs among these patients mostly arises from a deficiency of the apoptotic pathways in their lung cancer cells [38]. Accordingly, it is important to discover novel drugs with the ability to enhance lung cancer cell death via another type of programmed cell death.

Apoptosis and autophagy are evolutionarily conserved processes that regulate cell fate. However, the relationship between these two cellular processes is not yet to be well-defined. Studies have shown that despite the marked differences between these two processes, their regulation is somehow intimately connected and the same regulators can control both apoptosis and autophagy. In general, the PI3K/AKT/mTOR pathway is the major upstream pathway to inhibit autophagy and apoptosis [19]. Likewise, Bcl-2 was shown to be an important inhibitor for both autophagy and apoptosis. For autophagy, the inhibitory function of Bcl-2 occurs mainly at the endoplasmic reticulum (ER) site; however, Bcl-2 localization at mitochondria plays a key role in regulation of apoptosis [23,24]. Interestingly, we found that PE5 can suppress both the PI3K/AKT/mTOR pathway and Bcl-2, resulting in apoptosis and autophagic cell death. The previous study has shown that in certain conditions, apoptosis and autophagic cell death can be independent of each other [9]; we also found the similar phenomenon of PE5.

Generally, autophagy is a cellular process that is responsible for stress management and nutrient deprivation to ensure an adequate supply of the cellular basic units and bioenergy. In such a way, autophagy is recognized as a pro-survival mechanism. Previous evidence suggests that combination of autophagic inhibitor (wortmannin and chloroquine) and compound can use to sensitize cancer cell to chemotherapeutic drugs [39]. On the contrary, evidence has shown that a long period or excessive activation of autophagy can lead to cell destruction, termed “autophagic cell death (ACD)” or “type-II cell death” [40]. However, the conclusive mechanistic pathways involved in controlling ACD as well as the cellular decision for survival or death are largely unknown and, in most cases, they rely on several factors, including the duration and potency of autophagy induction and the cellular signaling condition [14,41]. As ACD is frequently observed as a backup cell death mechanism in drug-resistant or apoptosis-defective cancers [13,42], it may offer a new avenue for therapeutic exploitation. ACD has been shown to have significant value as a novel mechanism for cancer cell death induced by chemotherapeutics or natural compounds [41,43]. Previous studies have revealed that cellular pathways, including the adenosine monophosphate-activated protein kinase (AMPK) pathway and the phosphatidylinositol 3-kinase (PI3K)/AKT/mTOR pathway, function in the processes of autophagy and ACD [17,44]. A recent study found that inhibition of the AKT/mTOR axis is critical for the regulation of ACD in human NSCLCs [19]. Consistently, we found that PE5 exerts its ACD induction via suppression of the AKT/mTOR pathways (Figure 6).

PE5 induces ACD as a major mechanism of action through the suppression of AKT-mTOR and the anti-apoptotic members of the Bcl-2 family protein. In addition, the morphology of the PE5-treated cells displayed apoptosis characteristic including membrane blebbing and DNA condensation at 50–100 µM PE5, while the cells contained excessive vacuoles (Figure 1E, Figure 2A, and Figure 7B). Autophagosomes induced by PE5 exhibited a clear double membrane as observed by an electron microscope (Figure 2A,D and Figure 7B), with evidence from protein analysis showing the conversion of LC3-I to LC3-II (Figure 2E and Figure 3A,B). In addition, PE5 exhibited autophagic flux in H460 cells (Figure 2F,G).

We confirmed the involvement of autophagy in death induction by co-treatment of the compound with wortmannin, and the results indicated that when autophagy was suppressed, the cell death caused by PE5 was interrupted (Figure 3C,D). Consistent with our data, previous research found that autophagic inhibition by wortmannin could decrease autophagy-dependent cell death in Iso-GNA treated cells. Previous research found that the level of Atg7 was increased in a concentration dependent manner during autophagy induction [9]. Consistent with our data, we found that PE5 increased Atg7 in H460 cells. Moreover, we confirmed that autophagy-related (ATG) proteins are involved in autophagy induced by PE5 by using siRNA (Figure 3E,F). Consistent with our data, previous research found that siATG proteins reduced the conversion of LC3 and attenuated colon cancer cell death [10].

Proteomics is the use of quantitative protein analysis to illustrate biological processes, including drug effects and disease processes [35]. In this study, the key proteins altered by the treatment at different times were monitored in order to verify the time-dependent cellular profiles in response to cellular events at 12 and 24 h after treatment. We observed autophagy induction effect of PE5 at 12 h, while the cell viability remained 100% versus the control, suggesting that the results at this time point may reflect mainly autophagy induction or upstream regulation of the cells. However, at 24 h, when cell death was significantly found, this point may reflect mainly the mechanism of cell death. Our analysis showed that PE5 directly or indirectly modulated several proteins functioning in apoptosis and the autophagy pathway (Figure 5). A previous study also used proteomics profiling to determine the effect of resveratrol, a stilbene compound, in combination with doxorubicin, showing that resveratrol effectively sensitized MCF-7 cells to cytotoxic therapy and that HSP27 inhibition enhanced the cytotoxicity of doxorubicin [45]. Among these PE5-modulated proteins, we also found proteins associated with several cellular events described in cancer cells, including apoptosis inhibition and drug resistance [46].

The mTOR protein acts as a central regulator of cell growth, proliferation, and survival [47]. Importantly, mTOR has been recognized as a key modulator of autophagy [17], and inhibition of this protein has been demonstrated to cause autophagy [19], while the induction of mTOR could reduce autophagy [48]. In terms of its mechanism, mTOR controls autophagy through the regulation of a protein complex composed of unc-51-like kinase 1 (ULK1), autophagy-related gene 13 (ATG13), and the focal adhesion kinase family-interacting protein of 200 kDa (FIP200) through phosphorylating and inhibiting this kinase complex to initiate autophagy [49,50]. ULK1 is also known to directly phosphorylate the autophagy-related gene 14 (ATG14) in an mTOR-dependent manner, thus regulating ATG14 and the class-III PI3K vacuolar protein sorting 34 (VPS34) lipid kinase activity to control the level of autophagy [51]. Hence, these kinases represent attractive targets for therapeutic treatment involving autophagy.

From the STITCH database [52], the top 20 downregulated proteins in response to PE5 that had the most protein interactions were involved in the PI3K/AKT/mTOR pathway at 12 and 24 h (Figure 6A,B). Furthermore, we confirmed the proteomics results by using western blot analysis, which indicated the main mechanism of PE5 in mediating autophagy was though mTOR suppression. For cell death, we analyzed the crosstalk between apoptosis and autophagy to assess their co-relation. We found that the suppression of AKT-dependent proteins was observed at 12 h and sustained until 24 h of treatment, while the downregulation of mTOR-related proteins was found only at 12 h, suggesting that the suppression of AKT was responsible for mTOR inhibition at 12 h and may be involved in cell death at 24 h. Interestingly at 24 h, the depletion of Bcl-2 and Mcl-1 was noted, suggesting that ACD induced by PE5 may at least in part be related to Bcl-2 and Mcl-1 suppression. Regarding the effect of Bcl-2 on ACD, a previous study reported that the degradation of Bcl-2 was related to ACD and apoptosis [53]. Beclin-1 functioning in the process of autophagy was shown to be antagonized by Bcl-2 and other Bcl-2 family members, including Bcl-XL and Mcl-1 [54,55]. The role of Bcl-2 depletion on ACD induction was previously demonstrated in breast cancer cell lines. SiRNA-mediated Bcl-2 decrease was shown to induce ACD in MCF-7 cells [21]. Likewise, Mcl-1 depletion was shown to involve the induction of ACD by SC-59, a novel sorafenib derivative, in hepatocellular carcinoma cells [56].

The link between autophagy and apoptosis as apoptosis potentiator has been previously reported [10]. On the contrary, several studies have revealed the phenomenon that autophagy and apoptosis can be independent [9,11]. Likewise, our results showed that PE5 induced autophagic cell death independent of apoptosis. Apoptosis inhibitor (Z-VAD-FMK) could inhibit PE5-induced PARP cleavage, and the autophagy inhibitor failed to inhibit apoptosis (Figure 3G). Consistent with previous research data, oxyresveratrol increased cell death via ACD, which was independent of apoptosis induction. In addition, the oxyresveratrol effects were owing to changes in the activity levels of p38 MAPK and PI3K/AKT/mTOR [57].

**Figure 8:**
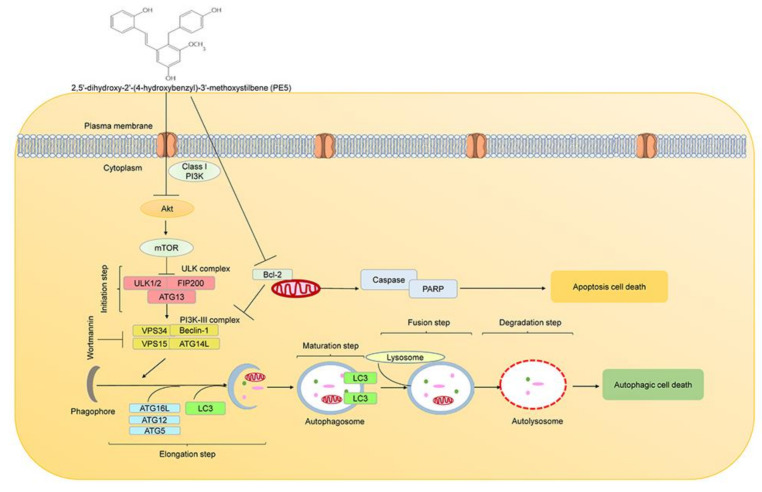
Schematic display of PE5 inducing autophagic cell death and apoptotic cell death.

Previous research have shown that natural compounds from the orchid exert potential pharmacological activities [26,28]. In this study, we expanded such knowledge toward their novel activity in ACD regulation. PE5, polyphenol compound, isolated from the roots of *Paphiopedilum exul.*, was demonstrated to facilitate ACD and apoptosis in lung cancer cells, with the underlying mechanisms presented in Figure 8. Polyphenols have an affinity for lipophilic compartment, which should locate in the cell membranes; however, the balance between the lipophilic and hydrophilic part of the compounds may integrate the possible localization of the compound, and not all polyphenols could solely stay in the cell membrane and interact with vital protein producing numerous regulatory effects [58]. Additionally, a number of studies have indicated the specific targets of polyphenol compounds in the cytoplasmic compartment of the cells [59,60]. Interestingly, we found that PE5 directly inhibited both PI3K/AKT/mTOR pathway, and Bcl-2 resulted in apoptosis and autophagic cell death (Figure 5 and Figure 6).

Stilbenoids are hydroxylated derivatives of stilbene, and the presence of hydroxyl group makes them superior antioxidant activities [61,62,63]. One of the recognized stilbenoid compounds is resveratrol, known for its anticancer activity against many cancer types, and the pathways involved are cell specific and concentration dependent. Recently, research found that resveratrol induced the apoptosis and ACD in A549 cells via a p53-dependent pathway [64]. Moreover, ACD induced by resveratrol depends on the Ca^2+^/AMPK/mTOR pathway [65]. In addition, resveratrol stimulates ACD in prostate cancer cells via down-regulation of STIM1 and the mTOR pathway [66]. Recently, a review reported that polyphenols are organic chemicals that not only exhibit potential antioxidant, but can also initiate the process of apoptosis and the extensive ACD in cancer cells [67]. Here, we found that although autophagy and apoptosis both contributed to cell death, they were not inter-dependent. These data propose the potential of PE5 as an anticancer approach and lead to a better understanding of ACD regulation, revealing AKT/mTOR as well as Bcl-2 suppression as therapeutic targets for driving cancer cells to autophagy-dependent cell death.

## 5. Conclusions

In conclusion, the data from this study established that PE5, polyphenol stilbene compound, can induce both autophagic cell death and apoptosis in NSCLCs by targeting AKT/mTOR and Bcl-2 suppression, which is the novel mechanism of PE5 in shifting survival role of autophagy toward cell death induction. This evidence may value and encourage the further investigation of this useful compound to be used for anti-cancer approaches and beneficial for improving the response to conventional drugs.

## Data Availability

The data presented in this study are available on request from the corresponding author.

## Supplementary Materials

The following are available online at https://www.mdpi.com/article/10.3390/antiox10040534/s1, Figure 1L: GAPDH; Figure 1J: PARP/Cleaved-PARP; Figure 2E: LC3; Figure 2F: GAPDH; Figure 3A: ATG5; Figure 3A: ATG7; Figure 3B: p62; Figure 6C. P-mTOR/Akt; Figure 6C: Bcl2; Figure 6C: PI3K; Figure 6C: Bax.
