# Supplementary material for: Targeting AKT/mTOR and Bcl-2 for Autophagic and Apoptosis Cell Death in Lung Cancer: Novel Activity of a Polyphenol Compound
Source: Antioxidants (Basel). 2021 Mar 29;10(4):534. doi: 10.3390/antiox10040534 (PMC8066183; doi:10.3390/antiox10040534)

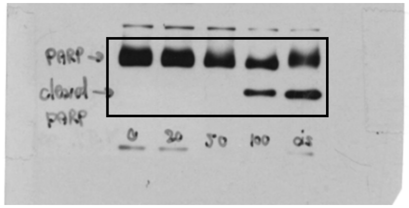

Fig 1J. PARP/Cleaved-PARP

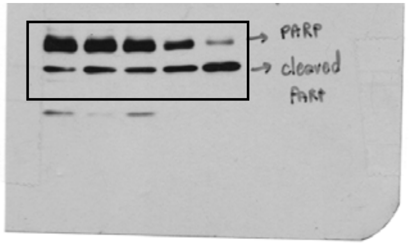

Fig 1L. PARP/Cleaved-PARP

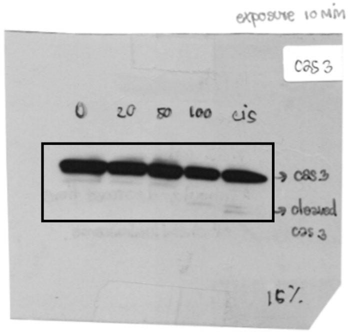

Fig 1J. Caspase3/cleaved caspase3

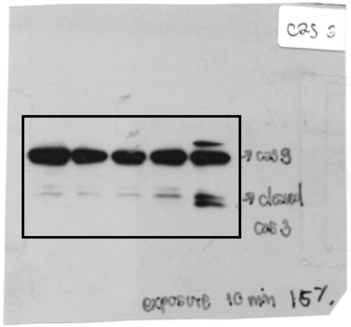

Fig 1L. Caspase3/cleaved caspase3

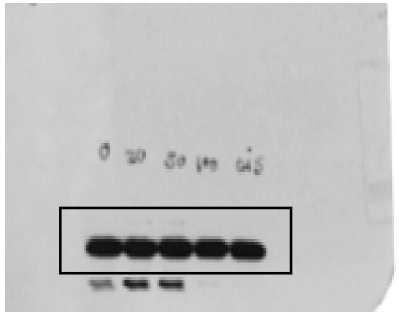

Fig 1J. GAPDH

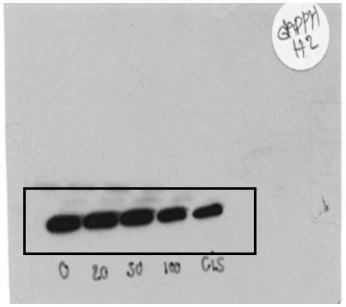

Fig 1L. GAPDH

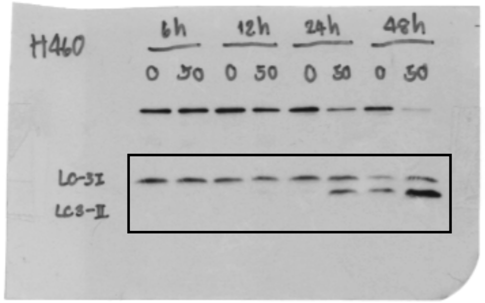

Fig 2E. LC3

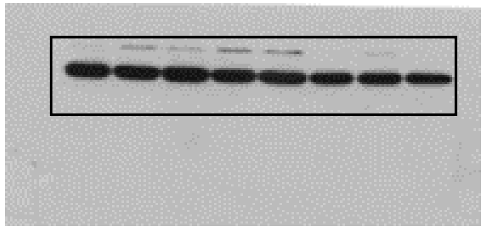
Fig 2E. GAPDH

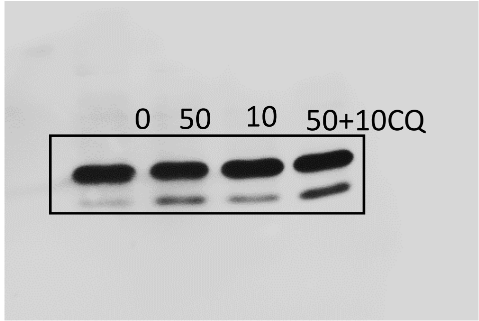

Fig 2F. LC3

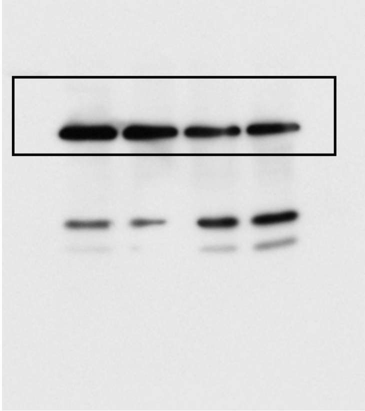
Fig 2F. GAPDH

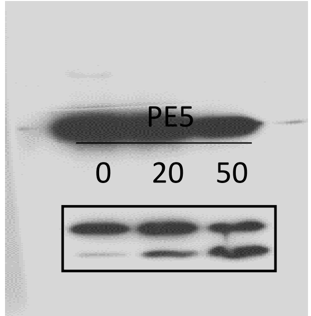

Fig 3A. LC3

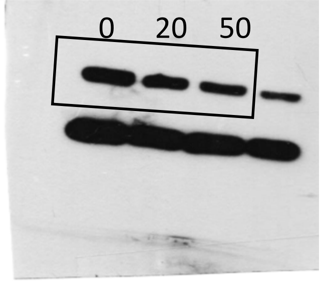

Fig 3A. ATG5

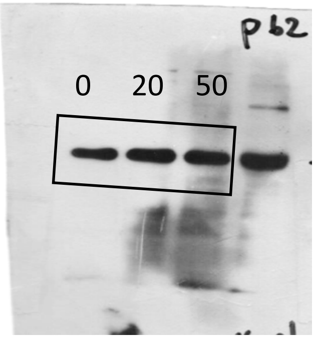

Fig 3A. p62

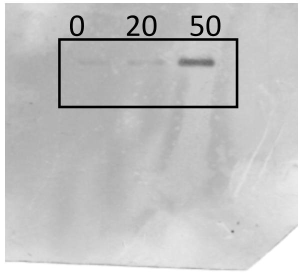

Fig 3A. ATG7

Fig 3A. GAPDH

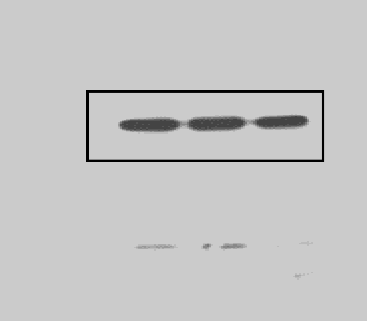

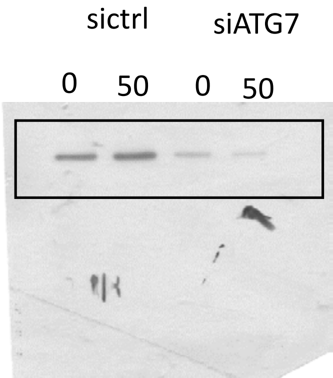

Fig 3E. ATG7

Fig 3E. LC3

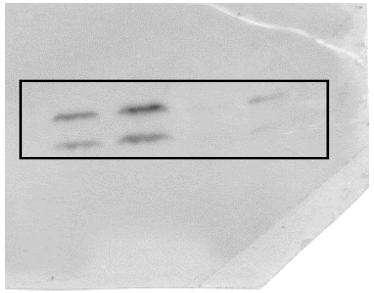

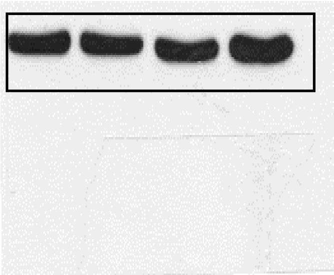
Fig 3E. GAPDH

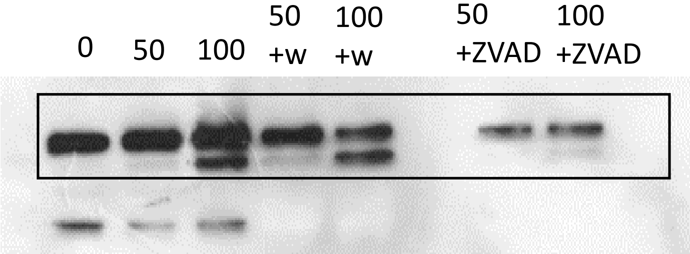

Fig 3G. PARP

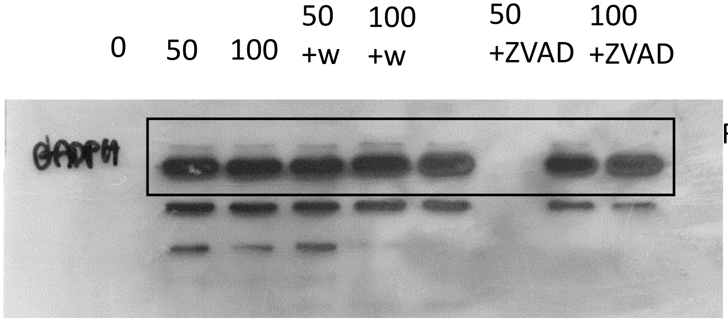

Fig 3G. GAPDH

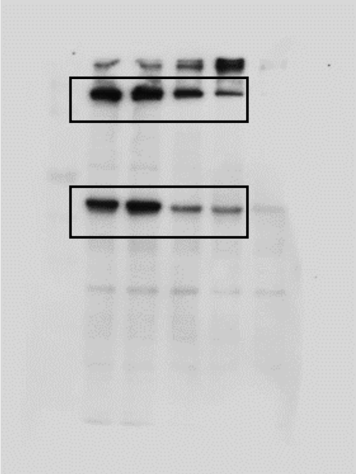

Fig 6C. P-mTOR/Akt

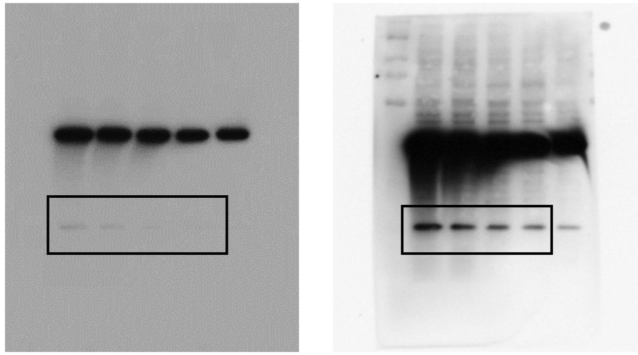

5 min

10 min

Fig 6C. Bcl2

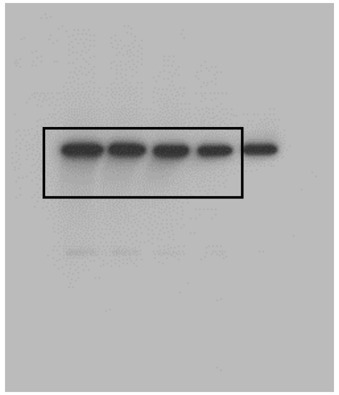

Fig 6C. GAPDH

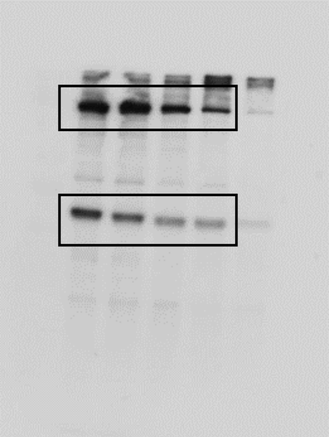

Fig 6C. mTOR/p-Akt

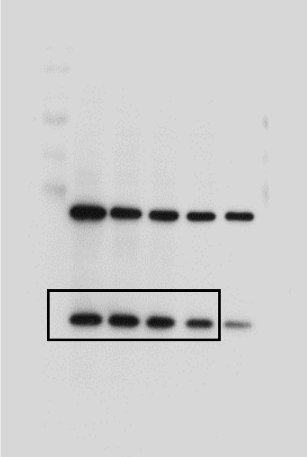

Fig 6C. Bax

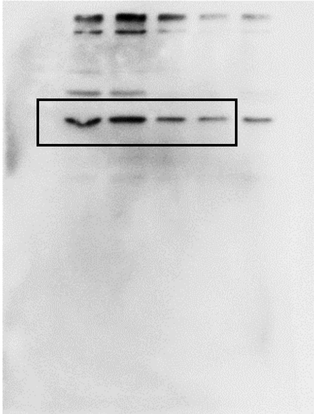

Fig 6C. PI3K

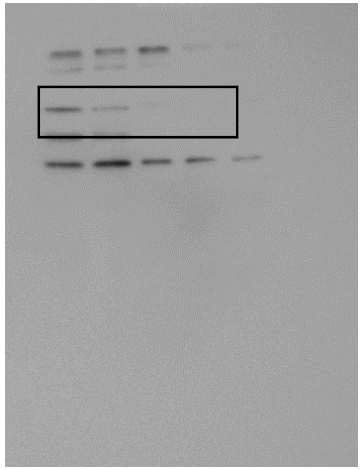

Fig 6C. P-PI3K

The purity of PE5

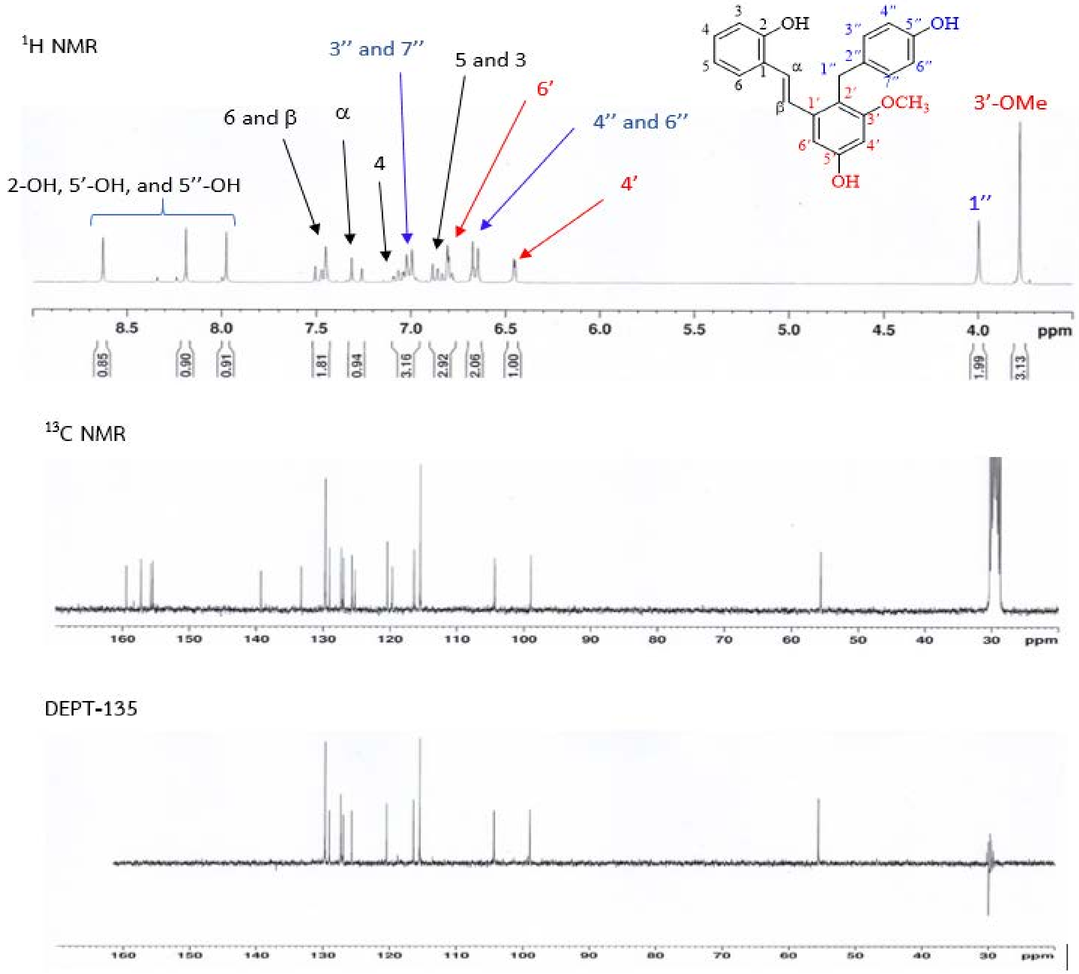

Supplement: Supplementary file 1 [file antioxidants-10-00534-s001.pdf]
